# Modeling and Compensation for Asymmetrical and Dynamic Hysteresis of Piezoelectric Actuators Using a Dynamic Delay Prandtl–Ishlinskii Model

**DOI:** 10.3390/mi12010092

**Published:** 2021-01-16

**Authors:** Wen Wang, Fuming Han, Zhanfeng Chen, Ruijin Wang, Chuanyong Wang, Keqing Lu, Jiahui Wang, Bingfeng Ju

**Affiliations:** 1 School of Mechanical Engineering, Hangzhou Dianzi University, Hangzhou 310018, China; wangwn@hdu.edu.cn (W.W.); hfm1810017@163.com (F.H.); wangrjcn@hdu.edu.cn (R.W.); cywang@hdu.edu.cn (C.W.); lkq@hdu.edu.cn (K.L.); wangjiahui20200119@163.com (J.W.); 2 State Key Laboratory of Fluid Power Transmission and Control, Zhejiang University, Hangzhou 310027, China; mbfju@zju.edu.cn

**Keywords:** piezoelectric actuator, dynamic hysteresis, asymmetrical hysteresis, Prandtl–Ishlinskii, hysteresis compensation

## Abstract

Piezoelectric actuators are widely used in micro- and nano-manufacturing and precision machining due to their superior performance. However, there are complex hysteresis nonlinear phenomena in piezoelectric actuators. In particular, the inherent hysteresis can be affected by the input frequency, and it sometimes exhibits asymmetrical characteristic. The existing dynamic hysteresis model is inaccurate in describing hysteresis of piezoelectric actuators at high frequency. In this paper, a Dynamic Delay Prandtl–Ishlinskii (DDPI) model is proposed to describe the asymmetrical and dynamic characteristics of piezoelectric actuators. First, the shape of the Delay Play operator is discussed under two delay coefficients. Then, the accuracy of the DDPI model is verified by experiments. Next, to compensate the asymmetrical and dynamic hysteresis, the compensator is designed based on the Inverse Dynamic Delay Prandtl–Ishlinskii (IDDPI) model. The effectiveness of the inverse compensator was verified by experiments. The results show that the DDPI model can accurately describe the asymmetrical and dynamic hysteresis, and the compensator can effectively suppress the hysteresis of the piezoelectric actuator. This research will be beneficial to extend the application of piezoelectric actuators.

## 1. Introduction

Due to the advantages of small thermal deformation, large driving force and fast frequency response [1,2], piezoelectric actuators are widely applied as micro-drive devices in atomic force microscopes [3,4], ultra-precision machine tools [5,6] and vibration control [7,8]. Although the piezoelectric actuator has many advantages, its material properties, creep [9,10], hysteresis [11,12] and other factors affect its positioning accuracy. In particular, the hysteresis characteristics have a great influence on the positioning accuracy of the piezoelectric actuator [13]. The inherent hysteresis of piezoelectric actuators are similar to other smart actuators such as magnetostrictive actuators [14,15] and shape memory alloy actuators [16], mainly referring to the nonlinearity between the input signal and its output signal [17]. The positioning error caused by the hysteresis may reach 15% [18], and this hysteresis is affected by the input frequency [19,20,21,22]. As the input frequency increases, the output is increasingly affected by the dynamic hysteresis of piezoelectric actuators, which makes the hysteresis more complicated and difficult to be described. In addition, the hysteresis nonlinearity may exhibit asymmetrical characteristic at high frequency. Therefore, it is necessary to study the asymmetrical and dynamic characteristics of piezoelectric actuators.

Feedforward control is one of several common methods of compensating piezoelectric actuators [23]. It mainly means to establish an inverse model that can accurately describe the hysteresis and use it as a compensator to control the piezoelectric actuators. Many rate-independent hysteresis models have been proposed to describe the static hysteresis of piezoelectric actuators. These hysteresis models include Jiles–Atherton model [24], Preisach model [25,26,27], Prandtl–Ishlinskii (PI) model [28,29,30], Krasnosel’skii–Pokrovskii (KP) model [31], etc. The Jiles–Atherton model is a mechanistic model, which is formed by an ordinary differential equation formulation. It is usually applied to a particular material. Preisach model, PI model and KP model are phenomenological models formed by the weighted integrals of different classical operators. In addition, some scholars have proposed other hysteresis models for piezoelectric materials, such as Gibbs energy calculation model [32], 3D constitutive model [33] and simplified micromechanical model [34]. Unfortunately, these classical hysteresis models cannot be used to describe the dynamic hysteresis characteristics of piezoelectric actuators. 

To describe the dynamic hysteresis characteristic of piezoelectric actuators, many researchers have also proposed modified rate-dependent hysteresis models. Al Janaideh et al. [35] proposed a dynamic threshold method, so that the Prandtl–Ishlinskii model can be used to describe the dynamic hysteresis. Jiang et al. [36] proposed a modified Prandtl–Ishlinskii model based on two asymmetrical operators, which can separately describe the delayed ascending branch and descending branch. Qin et al. [37] added the input rate to the weight function to ensure that the weight can be dynamically updated with the input, which makes up for the inability of the Classic Prandtl–Ishlinskii (CPI) model to describe the dynamic hysteresis. Yu et al. [38] proposed a dynamic Preisach model by adding the rate of change of the input to the weight function. Zhu et al. [39] added the frequency factor and the asymmetry factor to the Bouc–Wen model and proposed a generalized Bouc–Wen model to describe the asymmetrical and dynamic hysteresis characteristics of piezoelectric actuators. However, these dynamic hysteresis models are inaccurate or complex in describing the hysteresis characteristics.

In this paper, a Dynamic Delay Prandtl–Iishlinskii (DDPI) model is proposed to accurately describe the asymmetrical and dynamic hysteresis of piezoelectric actuators. The delay coefficient is introduced into the classical Play operator to adapt to the asymmetrical and dynamic characteristics of piezoelectric actuators. The DDPI model is not the same as other dynamic models which introduce the rate of input or output into static models. The experimental results show that the DDPI model can describe the asymmetrical and dynamic characteristics of the piezoelectric actuator accurately.

The remainder of this paper is organized as follows. Section 2 proposes the DDPI model and analyzes the roles of the rising delay coefficient and the falling delay coefficient. Section 3 introduces the RDPI model and verifies that the DDPI model is more accurate than the RDPI model. In Section 4, an inverse compensator is designed based on the Inverse Dynamic Delay Prandtl–Ishlinskii (IDDPI) model. The inverse compensator was verified to effectively suppress the dynamic asymmetry of the piezoelectric actuator by experiments, and the experimental results are discussed in Section 5. Finally, the conclusion is given in Section 6.

## 2. Hysteresis Model

The dynamic hysteresis characteristic has a greater impact on the positioning accuracy of piezoelectric actuators, especially when high-frequency signal is used to drive the piezoelectric actuators. Therefore, it is necessary to establish a dynamic hysteresis model to describe the dynamic characteristic of piezoelectric actuators. In this section, the CPI model and the DDPI model are introduced in brief, and the roles of the new parameters on the operator shape are analyzed.

### 2.1. Classic Prandtl–Ishlinskii Model

The CPI model is a common operator type hysteresis model. CPI model is established by the Play operator. The Play operator is as follows:

(1) $$\left\{ \begin{array}{l} F_{r}(k)=\max(u(k)-r,\min(u(k)+r,F_{r}(k-1)))\\ F_{r}(0)=\max(u(0)-r,\min(u(0)+r,0)) \end{array}\right.$$

where $F_{r}(k)$ is the operator output at the *k*th moment, $F_{r}(k-1)$ is the operator output at the (*k*-1)th moment, u(k) is the input signal at the *k*th moment, r is the threshold of the operator, u(0) is the initial input signal value and $F_{r}(0)$ is the initial operator value.

CPI model is a hysteresis model formed by superposition of multiple Play operators, as follows:

(2) $$y(k)=p_{0}u(k)+\sum_{i=1}^{n}p_{r}(r_{i})F_{r_{i}}(k)$$

where y(k) is the output signal at the *k*th moment, $p_{0}>0$ is the linear coefficient, $p_{r}(r_{i})$ is the weight of the operator and *n* is the length of the input signal. The weight of the operator can be identified by the algorithm and is generally greater than zero.

### 2.2. Dynamic Delay Prandtl–Ishlinskii Model

The CPI model cannot describe the dynamic and asymmetric hysteresis. It is difficult to eliminate the influence of the asymmetrical and dynamic characteristics of the piezoelectric actuator on the positioning accuracy of the system when the CPI model is used as a compensator to suppress the hysteresis characteristics of the piezoelectric actuator.

#### 2.2.1. Modified Model

Therefore, it is necessary to modify the CPI model and the Play operator. The modified Delay Play operator is as follows:

(3) $$F_{r_{i},\tau ,\varphi }[u](k)=\max\left\{u(k-\varphi )-r_{i},\min\left\{u(k-\tau )+r_{i},F_{r_{i},\tau ,\varphi }[u](k-1)\right\}\right\}$$

where $F_{r_{i},\tau ,\varphi }[u](k)$ is the output of the Delay Play operator at the *k*th moment, $F_{r_{i},\tau ,\varphi }[u](k-1)$ is the output of the Delay Play operator at the (*k − 1*)th time, τ is the rising delay coefficient and φ is the falling delay coefficient. The rising and falling delay coefficients should be greater than zero.

The DDPI model is defined as:

(4) $$y(k)=p_{0}u(k)+\sum_{i=1}^{n}p_{r}(r_{i})F_{r_{i},\tau ,\varphi }[u](k)$$

where $p_{0}$ is the linear coefficient of the memoryless function and $p_{r}(r_{i})$ is the weight of the operator.

#### 2.2.2. The Role of Two Delay Coefficients

To explore the influence of the two delay coefficients on the shape of the Delay Play operator, simulations were carried out. The input signal is *u*(*t*) = 5sin(2*πft*), the threshold is *r =* 2 and the sampling frequency is *T =* 100 k Hz.

**Figure 1:**
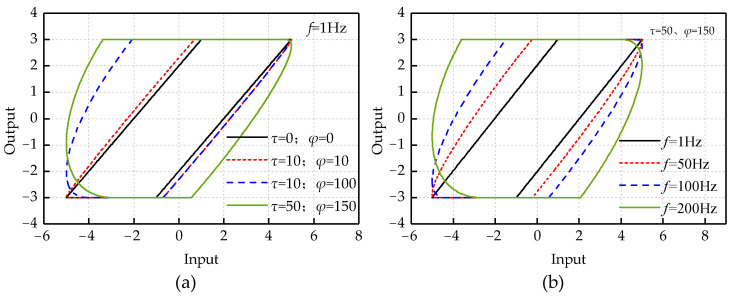
The role of two delay coefficients: (**a**) at the same frequency, the relationship between the shape of operator and the delay coefficient; and (**b**) at the same delay coefficient, the relationship between the shape of operator and frequency.

Figure 1 shows the influence of *τ* and *φ* on the shape of the Delay Play operator in the two cases. The first case is to analyze the influence of the change of delay coefficient on the shape of Delay Play operator when the input signal is same. The second case is to explore how the shape of the Delay Play operator is affected by the input frequency changes when the delay coefficient is consistent. The values of *τ* and *φ* are controlled within 200.

Figure 1a shows that, at the input frequency *f* = 1 Hz, the width of the Delay Play operator increases with the increase of *τ* and *φ*. The rising and falling edges of the Delay Play operator change from a straight line to an arc, and the curvature of the inflection point of the operator curve increases with the increase of *τ* and *φ*. As shown in Figure 1b, with the increase of the input frequency, the width of Delay Play operator is also increasing when *τ* and *φ* remain the same.

Through the simulations, the shapes of the two curves are affected by input frequency similarly. One is the influence of input frequency on the shape of the Delay Play operator when the rising delay coefficient and the falling delay coefficient are not zero. The other is the influence of the input–output relationship of the piezoelectric actuator with the input frequency.

## 3. Experiment Verification

To verify the performance of the DDPI model for describing the asymmetrical and dynamic hysteresis characteristics of piezoelectric actuators, it is necessary to acquire experiment data by comparison experiments. This section introduces the experimental setup and analyzes the model comparison results in brief.

### 3.1. Experimental Setup

**Figure 2:**
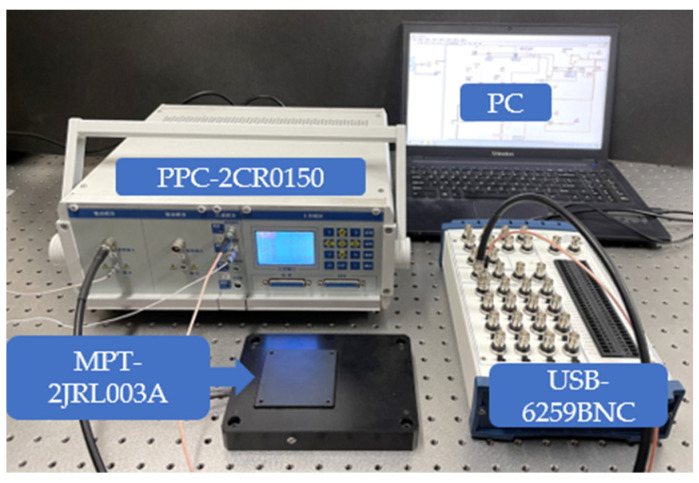
Schematic diagram of experimental equipment.

Figure 2 shows the experimental setup. The piezoelectric two-dimensional positioning platform MPT-2JRL003A has a displacement range of 55 μm × 55 μm, a displacement resolution of 5 nm and a repeat positioning accuracy of 50 nm. The study only carried out experiments on the one-dimensional hysteresis of the *x*-axis. The precision positioning controller PPC-2CR0150 has a sensor module with a sensitivity of 3 μm/V and a drive module with a gain of 0-10. The data acquisition system includes a data acquisition card and PC that can use the LabVIEW software. The data acquisition card is National Instruments USB-6259BNC, which has 32 channels of 16-bit sampling rate of 1.25 MS/s analog input channels and 4 channels of 16-bit sampling rate of 2.8 MS/s analog output channels.

### 3.2. Experiment Design

**Figure 3:**
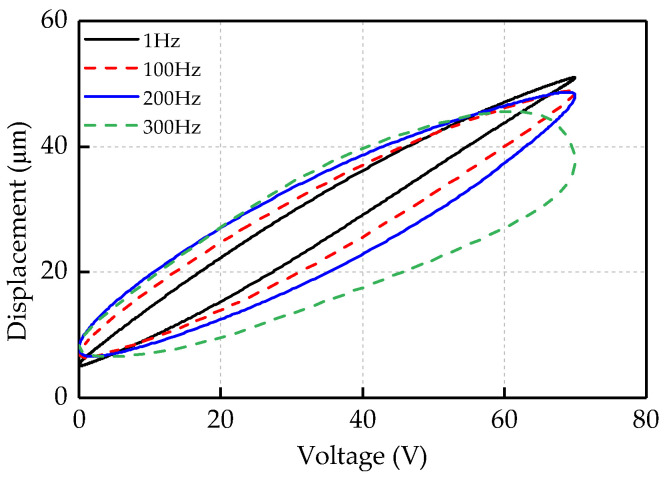
Voltage–displacement relationship at different frequencies.

To verify the validity and accuracy of the model, it is necessary to acquire experiment data for verification and comparison. The input signal of *u*(*t*) = 35sin(2*πft −* *π*/2) is used to drive the *x*-axis of piezoelectric platform to obtain the displacement signal data, and the value range of input frequency is 1–300 Hz. Figure 3 shows the voltage–displacement relationship from a part of experiments.

By predicting the displacement of the platform, the performance of the DDPI model and the Rate-Dependent Prandtl–Ishlinskii (RDPI) model [35] to describe the hysteresis of the dynamic and asymmetry of the piezoelectric actuator is analyzed.

To evaluate the performance of the two models which predict the displacement of the piezoelectric actuator, four different evaluation are utilized, namely Maximum Absolute Error (MAE), Maximum Relative Error (MRE), Root Mean Square Error (RMSE) and E_ratio_ [40], which are defined as follows:

(5) $$\mathrm{MAE}=\max_{k\in [1,n]}\left|y(k)-Y(k)\right|$$

(6) $$\mathrm{MRE}=\mathrm{MAE}/y_{\max}\times 100\%$$

(7) $$\mathrm{RMSE}=\sqrt{\frac{1}{n}\sum_{k=1}^{n}{\left|y(k)-Y(k)\right|}^{2}}$$

(8) $$E_{\mathrm{ratio}}=\frac{\sum_{k=1}^{n}\left|y(k)-Y(k)\right|}{\sum_{k=1}^{n}\left|y(k)-y_{\mathrm{mean}}\right|}$$

where *n* is the length of the displacement signal; y(k) is the experimental displacement at the *k*th moment; Y(k) is the model displacement at the *k*th moment; $y_{\max}$ is the maximum value of the experimental displacement signal; and $y_{\mathrm{mean}}$ is the average value of the experimental displacement signal.

The RDPI model is defined as (to describe the asymmetry of piezoelectric actuator, the original single dynamic threshold is changed to double dynamic thresholds):

(9) $$\left\{ \begin{array}{l} F_{r_{i}}[u](k)=\max\left\{u(k)-r\prime [\dot{u}](k),\min\left\{u(k)-r\prime \prime [\dot{u}](k),F_{r_{i}}[u](k-1)\right\}\right\}\\ y(k)=p_{0}u(k)+\sum_{i=1}^{n}p_{r}(r_{i})F_{r_{i}}[u](k)\\ r^{\prime }[\dot{u}](k)=r+\alpha \left|\dot{u}(k)\right|\\ r^{\prime \prime }[\dot{u}](k)=r+\beta \left|\dot{u}(k)\right| \end{array}\right.$$

where $F_{r_{i}}[u](k)$ is the operator output of the RDPI model at the *k*th moment; $F_{r_{i}}[u](k-1)$ is the operator output of the RDPI model at the (*k −* 1)th moment; $r^{\prime }[\dot{u}](k)\;\mathrm{and}\;r^{\prime \prime }[\dot{u}](k)$ are the dynamic threshold of the rising and falling edges of the operator at the *k*th moment, respectively; $\dot{u}(k)$ is the rate of input signal at the *k*th moment; *α* and *β* are positive constants; $p_{0}$ is the linear coefficient of the memoryless function; and $p_{r}(r_{i})$ is the weight of the operator. Both the linear coefficient of the memoryless function and the weight of the operator are identified by Differential Evolution (DE) algorithm.

To verify the performance of the two models to describe the asymmetrical and dynamic hysteresis characteristics of piezoelectric actuators, two kinds of comparative experiments were carried out, including seven groups of single frequency signal experiments and two groups of multi-frequency signal experiments.

Experiment 1: Set the input voltage as *u*(*t*) = 35sin(2*πft* − *π*/2) + 35. The frequency is {1, 50, 100, 150, 200, 250, 300} Hz. The threshold is set to {0, 7, 14, 21, 28, 35, 42, 49, 56, 63}. The weights and linear coefficients of two models are identified by DE algorithm.

Experiment 2: Set the input voltage as *u*_1_(*t*) = 10[sin(100*πt* − *π*/2) + sin(300*πt* − *π*/2) + sin(500*π t* − *π*/2) + 3] and *u*_2_(*t*) = 10[sin(400*πt* − *π*/2) + sin(500*πt* − *π*/2) + 2]. *u*_1_(*t*) and *u*_2_(*t*) are two different multi-frequency signals. The frequencies of two signals contain are {50, 150, 250} Hz and {200,250} Hz, respectively. The threshold is set to *r*_1_ = {0,6,12,18,24,30,36,42,48,54} and *r*_2_ = {0,4,8,12,16,20,24,28,32,36}.

### 3.3. Experiment Results

**Figure 4:**
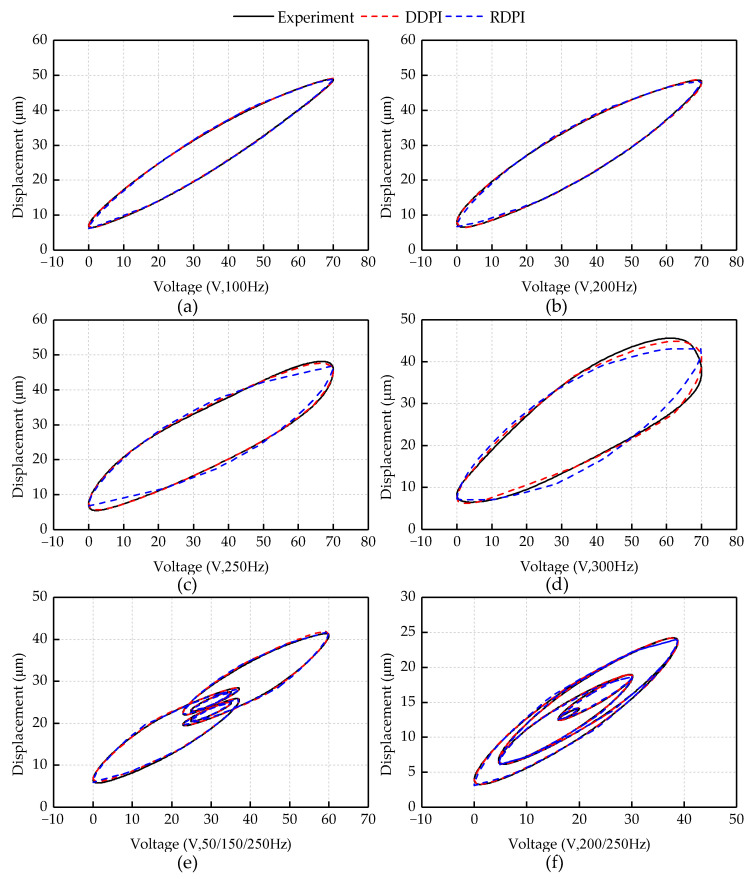
Comparison of DDPI and RDPI models with experimental data at frequencies: (**a**) 100 Hz; (**b**) 200 Hz; (**c**) 250 Hz; (**d**) 300 Hz; (**e**) 50/150/250 Hz; and (**f**) 200/250 Hz.

Figure 4 shows the fitting performances of the DDPI model and RDPI model on different experimental curves. 

**Figure 5:**
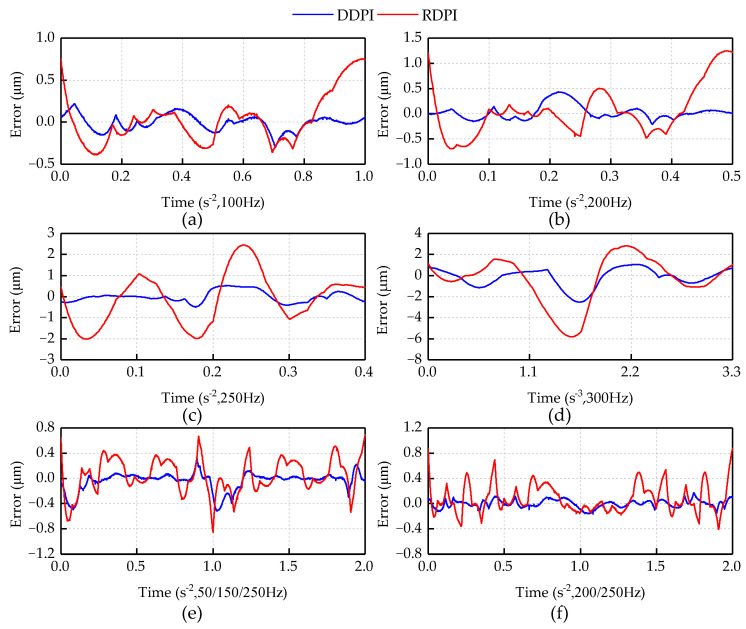
Error of two models at frequencies: (**a**) 100 Hz; (**b**) 200 Hz; (**c**) 250 Hz; (**d**) 300 Hz; (**e**) 50/150/250 Hz; and (**f**) 200/250 Hz.

To compare the fitting performances of the two hysteresis models to different experimental curves more clearly, Figure 5 shows the fitting errors of the two hysteresis models. In the model fitting of the 100 and 200 Hz single frequency experimental curves and the first multi-frequency experimental curve, the two hysteresis models describe the asymmetrical and dynamic characteristics of the piezoelectric actuator accurately. In the model fitting of the 250 and 300 Hz single frequency experimental curves and the second multi-frequency experimental curve, the fitting performance of the RDPI model is worse than that of the DDPI model. It can be found from the fitting conditions that the RDPI model has insufficient description of the higher frequency dynamic hysteresis curve. This is mainly reflected in the insufficient description of the inflection point of the hysteresis curve by the RDPI model. The RDPI model presents the straight-arc curve, which is inconformity with the arc of the inflection point on the high frequency hysteresis curve. When predicting different experimental displacements, the performance of the DDPI model is mostly consistent. In the comparison of two groups of multi-frequency experiments, it can be clearly found that the DDPI model has a better performance to describe the minor loop, while the RDPI model still has a larger error in describing the minor loop.

**Figure 6:**
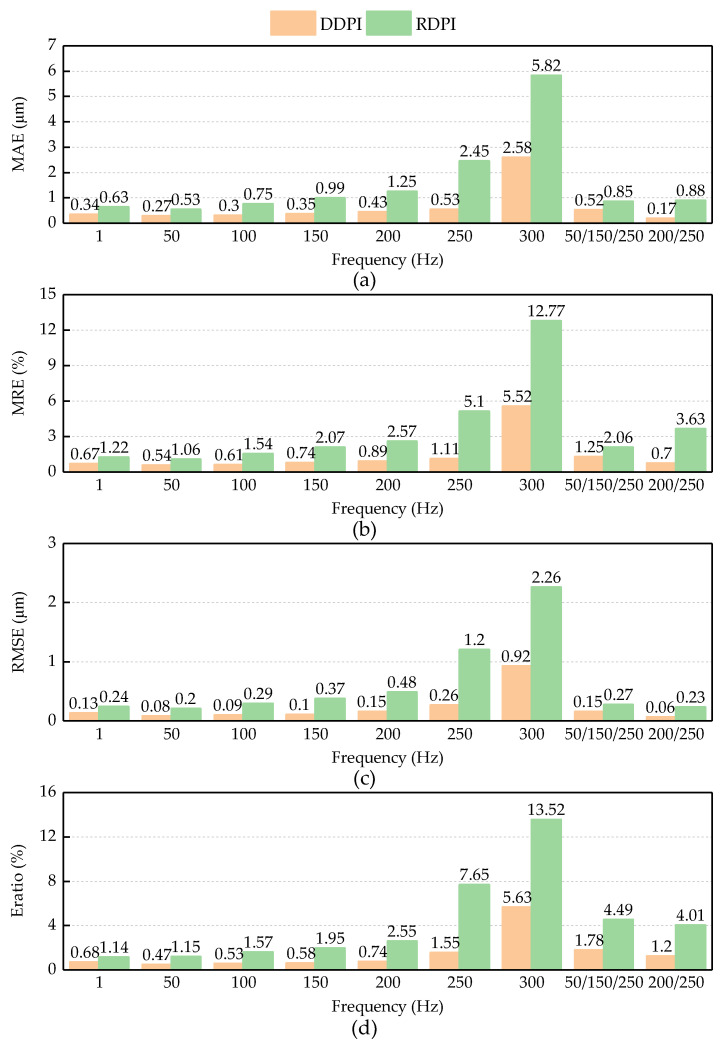
Evaluation of DDPI and RDPI error parameters: (**a**) MAE; (**b**) MRE; (**c**) RMSE; and (**d**) E_ratio_.

Figure 6 shows the evaluation results of the two models. The two hysteresis models can describe the hysteresis curve accurately at low frequency. The MRE of the DDPI model is less than 1% at 1–200 Hz and second multi-frequency experiments. When the frequency of the single frequency experiment is 250 Hz, the MRE of the DDPI model is less than 2%. The MRE of the DDPI model is less than 2% in the first multi-frequency experiment. The MRE of the RDPI model is more than 1% in the nine kinds of experiments. After the frequency of input signal is greater than 100 Hz, the MRE of the RDPI model exceeds 2%, and the description error is up to about five times compared with the DDPI model. However, when the input frequency is 300 Hz, the performance of the DDPI model decreases. However, the MRE of the DDPI model is still less than half that of RDPI models with MRE over 10%. It can be seen from different evaluation standards that the accuracy of the RDPI models decreases as the frequency increases on the whole. The DDPI model has a consistent performance on predicting different experimental displacements and the MAE of the DDPI model is less than 0.6 μm except 300 Hz. The RMSE of the DDPI model is less than 1 μm. However, the RMSE of the RDPI model exceeds 250 μm at 1 Hz and exceeds 2 μm at 300 Hz. On describing the same experimental curve, the accuracy of the DDPI model is higher than that of the RDPI model and the error is basically reduced by more than 40%. 

## 4. Feedforward Control

To suppress the asymmetrical and dynamic hysteresis characteristics of piezoelectric actuators, it is necessary to establish an inverse hysteresis model for feedforward control of piezoelectric actuators. 

**Figure 7:**
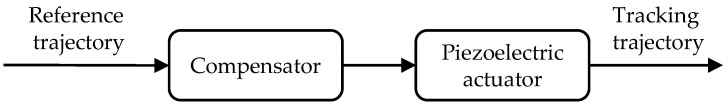
Schematic diagram of feedforward control.

The inherent hysteresis characteristics of piezoelectric actuator has an impact on the positioning accuracy of the system. To improve the positioning accuracy of the piezoelectric actuator, it is necessary to compensate the hysteresis of piezoelectric actuators. The common method is to establish an inverse compensator based on hysteresis model to perform feedforward control of the piezoelectric actuator. As shown in Figure 7, the inverse hysteresis model is a compensator to linearly control the tracking trajectory by inputting the reference trajectory.

Analytical inverse hysteresis model is one of several common inverse hysteresis models. However, after the Classic Prandtl–Ishlinskii model is modified to the Dynamic Delay Prandtl–Ishlinskii model, the operator is changed, which greatly increases the difficulty of deriving the analytical inverse hysteresis model. Compared with the analytical inverse hysteresis model, the inverse hysteresis model based on the Stop operator is simpler and more suitable for solving the IDDPI model. There is a definite relationship between the two operators, as follows:

(10) $$F_{r}(v)+E_{r}(v)=v$$

The relationship between the Stop operator and the Play operator provides assistance for establishing the IDDPI model. The Delay Stop operator of the IDDPI model is as follows

(11) $$E_{r,\tau ,\varphi }[y^{\prime }](k)=\min\left\{y^{\prime }(k)-y^{\prime }(k-\varphi )+r,\max\left\{\begin{array}{l} y^{\prime }(k)-y^{\prime }(k-\tau )-r,\\ y^{\prime }(k)-y^{\prime }(k-1)+E_{r,\tau ,\varphi }[y^{\prime }](k-1) \end{array}\right\}\right\}$$

where $E_{r,\tau ,\varphi }[y^{\prime }](k)$ is the output of the Delay Stop operator at the *k*th moment, $E_{r,\tau ,\varphi }[y^{\prime }](k-1)$ is the output of the Delay Stop operator at the (*k −* 1)th moment, $y^{\prime }(k)$ is the input signal of the IDDPI model at the *k*th moment and $y^{\prime }(k-1)$ is the input signal of the IDDPI model at the (*k − 1*)th moment.

The IDDPI model is formed by the Delay Stop operator and the memoryless function, as follows:

(12) $$u^{\prime }(k)={p^{\prime }}_{0}y^{\prime }(k)+\sum_{i=1}^{n}{p^{\prime }}_{r}(r_{i})E_{r_{i},\tau ,\varphi }[y^{\prime }](k)$$

where $u^{\prime }(k)$ is the output signal at *k*th moment, ${p^{\prime }}_{0}>0$ is the positive linear coefficient of the memoryless function and $p_{r}(r_{i})>0$ is the positive weight of operator.

## 5. Results and Discussion

### 5.1. Results

As the inverse hysteresis model for feedforward control of piezoelectric actuators, the expression of the IDDPI model is derived. To verify that the inverse compensator can effectively suppress the asymmetrical and dynamic hysteresis of piezoelectric actuators, two experiments with single frequency sinusoidal signals and multi-frequency sinusoidal signals were carried out. 

**Figure 8:**
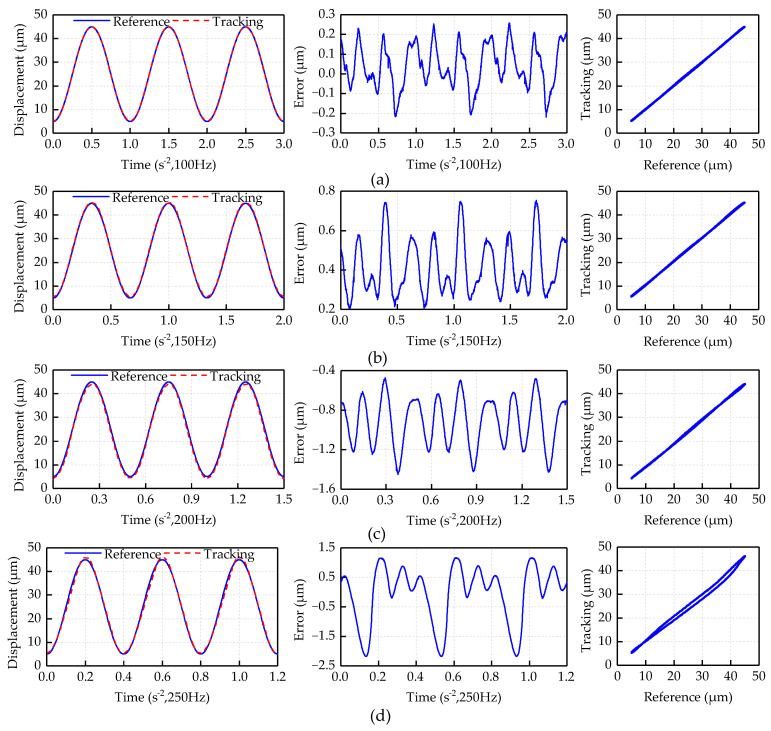
Feedforward experiment results of single frequency sinusoidal signal: (**a**) 1 Hz; (**b**) 100 Hz; (**c**) 200 Hz; and (**d**) 250 Hz.

Figure 8 shows the experimental results of feedforward compensation for the single frequency sinusoidal signals. The expected displacement is set as *y_d_*(*t*) = 20sin(2*πft* − *π*/2) + 25. The experimental results show that the inverse compensator can effectively suppress the asymmetrical and dynamic hysteresis of the piezoelectric actuator. The maximum absolute values of positioning error are 0.26, 0.75, 1.45 and 2.18 μm within 100, 150, 200 and 250 Hz, respectively. In the tracking experimental results with frequency of 100, 150 and 200 Hz, the reference trajectory and the tracking trajectory can mainly show a linear relationship. In the tracking experimental results with frequency of 250 Hz, the compensation performance of the inverse compensator has decreased. The nonlinear error of the 250 Hz experimental curve is reduced from 36% to 5.0% after compensation.

**Figure 9:**
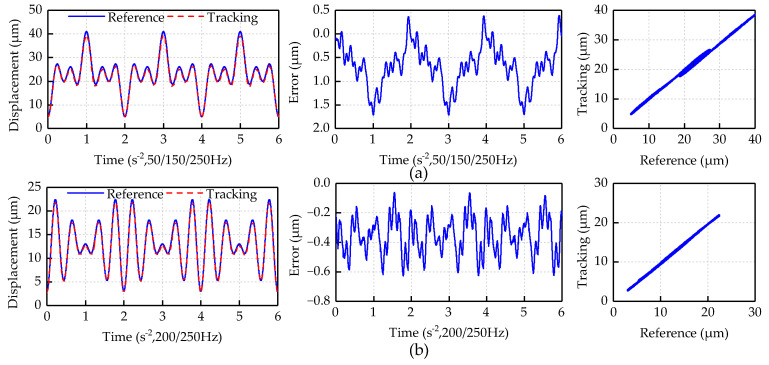
Feedforward experiment results of multi-frequency sinusoidal signal: (**a**) 50/150/250 Hz; and (**b**) 200/250 Hz.

Figure 9 indicates the experimental results of feedforward compensation for feedforward control driven by the multi-frequency sinusoidal signals. The reference trajectory is set as *y_d_*_1_(*t*) = 6[sin(100*πt* − *π*/2) + sin(300*πt* − *π*/2) + sin(500*πt* − *π*/2)] + 23 and *y_d_*_2_(t) = 5[sin(400*πt* − *π*/2) + sin(500*πt* − *π*/2)] + 13. The experimental results show that the inverse compensator can also effectively suppress the asymmetrical and dynamic hysteresis characteristics of the piezoelectric platform driven by the multi-frequency sinusoidal signal. The maximum absolute values of the positioning error driven by the two multi-frequency sinusoidal signals are 1.71 and 0.62 μm, respectively. In the first feedforward experiment of multi-frequency sinusoidal signal, the nonlinear error has decreased from 24% to 4.3%. In the second feedforward experiment of multi-frequency sinusoidal signal, the nonlinear error has decreased from 34% to 2%.

### 5.2. Discussion

Piezoelectric actuators are widely used in the fields of micromachining [41,42]. In the field of ultra-precision machining, there are higher requirements for the positioning accuracy of micro-position devices such as piezoelectric actuators. Due to the increase in frequency, the dynamic hysteresis characteristics of piezoelectric actuators have a greater impact on the positioning accuracy of the system [43,44].

Most dynamic hysteresis models describe the dynamic characteristic of piezoelectric actuators by introducing input or output rates to static models. However, the Dynamic Delay Prandtl–Ishlinskii model can describe both the asymmetrical and dynamic characteristics of piezoelectric actuators by introducing the rising and falling delay coefficients based on the Classical Prandtl–Ishlinskii model. This is not the same way as introducing the rate of input or output. With the change of the two delay coefficients, the shape of the operator can also be changed. Therefore, for most piezoelectric actuators, the Dynamic Delay Prandtl–Ishlinskii model can be relatively well predicted.

The above experimental results show that the Dynamic Delay Prandtl–Ishlinskii model has good accuracy hen fitting both low-frequency and high-frequency hysteresis curves of the piezoelectric actuator. For different input signals, the compensator based on the Inverse Dynamic Delay Prandtl–Ishlinskii model can effectively suppress the hysteresis of piezoelectric actuator.

**Figure 10:**
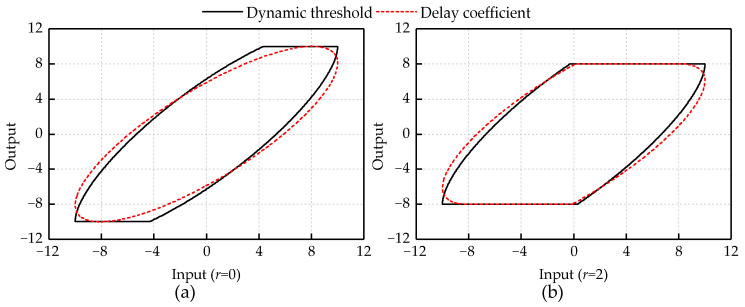
Comparison of two operators. (**a**) *r* = 0; and (**b**) *r* = 2.

Compared with the RDPI model, the DDPI model has a better performance on fitting the asymmetrical and dynamic hysteresis. In the above experimental results, it is found that the larger fitting error of the RDPI model is mainly concentrated in the inflection point of the experimental curve. To further explore the difference of the description performance of different models, two simulations of the Dynamic Threshold Play operator and the Delay Play operator are set up. The input signal is set as *u*(*t*) = 10sin(20*πt*) and the threshold is set as *r* = 0, 2. Figure 10 shows the results of two simulations. Under different thresholds, there is always a horizontal line at the top of the Dynamic Threshold Play operator, and there is a peak between the line and the arc. This is more difficult for the RDPI model to describe the smooth inflection point of experimental curve. When the threshold is 2, the inflection point of Delay Play operator also shows the shape of arc. Moreover, when the threshold is 0, the Delay Play operator presents the shape of an ellipse. These are reasons that the DDPI model can describe dynamic characteristics of the piezoelectric actuator accurately.

Although the DDPI model has a better performance on describing the dynamic asymmetry hysteresis, the accuracy of the DDPI model is obviously reduced in the fitting of the 300 Hz single frequency signal experimental curve. This may be because the experimental data are acquired by the piezoelectric platform, which has platform characteristics in addition to the hysteresis characteristics. The influence of the platform characteristics increases with the increase of frequency on displacement data. Therefore, it is difficult to predict the displacement of the piezoelectric platform using only the hysteresis model. It is necessary to add the dynamic model of the platform. Although in feedforward control, there is still the error between the reference trajectory and the tracking trajectory, when the frequency of signal is 250 Hz. The inverse compensator can mainly compensate the asymmetrical and dynamic hysteresis. It is helpful to improve the positioning accuracy of piezoelectric actuator and the working accuracy of ultra-precision machine tool.

## 6. Conclusions

The asymmetrical and dynamic hysteresis of piezoelectric actuators is difficult to describe and has an impact on application of piezoelectric actuators. This paper modifies the Classic Prandtl–Ishlinskii model and provides the expression of the Dynamic Delay Prandtl–Ishlinskii model that can describe the asymmetrical and dynamic characteristics of piezoelectric actuators at the same time. First, the influence of the rising and the falling delay coefficients is analyzed on the Delay Play operator. Then, the Dynamic Delay Prandtl–Ishlinskii model is compared with the existing dynamic hysteresis model. The experimental results show that the MAE of the Dynamic Delay Prandtl–Ishlinskii model is reduced by up to 80% compared with the Rate-Dependent Prandtl–Ishlinskii model. Finally, the inverse compensator is designed based on the Inverse Dynamic Delay Prandtl–Ishlinskii model, and the effectiveness of the inverse compensator was verified by several experiments. Experimental results show that the inverse compensator can effectively suppress the asymmetrical and dynamic hysteresis of piezoelectric actuators. This research is helpful to improve the position accuracy of piezoelectric actuators.
